# Model of an Open-Source MicroPython Library for GSM NB-IoT

**DOI:** 10.3390/s25175322

**Published:** 2025-08-27

**Authors:** Antonii Lupandin, Volodymyr Kopieikin, Maksym Khruslov, Iryna Artyshchuk, Ruslan Shevchuk

**Affiliations:** 1 Department of Computer Systems and Robotics, Educational and Scientific Institute of Computer Science and Artificial Intelligence, V. N. Karazin Kharkiv National University, 61022 Kharkiv, Ukraine; volodymyr.kopieikin@karazin.ua (V.K.); maksym.khruslov@karazin.ua (M.K.); 2 Department of Data Science, University of the National Education Commission, 30-084 Krakow, Poland; iryna.artyshchuk@uken.krakow.pl; 3 Department of Computer Science and Automatics, Faculty of Mechanical Engineering and Computer Science, University of Bielsko-Biala, 43-300 Bielsko-Biala, Poland; 4 Department of Computer Science, West Ukrainian National University, 46009 Ternopil, Ukraine

**Keywords:** MicroPython, NB-IoT, GSM modules, AT commands, IoT development, embedded systems, open-source software

## Abstract

The growing adoption of the Internet of Things (IoT) demands scalable, energy-efficient communication for autonomous devices. Narrowband IoT (NB-IoT), as a low-power wide-area technology, offers reliable connectivity but remains difficult to integrate in MicroPython systems due to the absence of high-level GSM libraries. This paper introduces a modular, object-oriented MicroPython library that abstracts AT command handling, automates network configuration, and supports protocols such as MQTT and Blynk. The architecture features a layered, hardware-agnostic core and device-specific adapters, enhancing portability and extensibility. The library includes structured exception handling and automated retries to improve system reliability. Empirical validation using a Raspberry Pi Pico and SIM7020E module in a typical IoT scenario demonstrated an up to 81% reduction in implementation time. By providing a reusable and extensible framework, this work improves developer productivity, enhances error resilience, and establishes a solid foundation for rapid NB-IoT application development. Future directions include cross-hardware validation and AI-assisted code and test generation.

## 1. Introduction

The exponential growth of the Internet of Things (IoT) is fundamentally transforming modern technology by enabling seamless data exchange and interaction between diverse devices and systems via the Internet [1,2,3]. This technological shift impacts multiple domains, including consumer electronics, healthcare, agriculture, environmental monitoring, and industrial automation, driving a need for efficient, robust, and cost-effective communication solutions. Among these, narrowband IoT (NB-IoT) has emerged as a key connectivity technology, particularly suitable for applications requiring low power consumption, extended coverage, and high reliability. NB-IoT, as a standard of low-power wide-area networks (LPWAN), utilizes existing cellular infrastructure to facilitate widespread deployment while minimizing operational costs [4,5,6,7,8,9,10,11,12].

While GSM modules supporting NB-IoT are increasingly prevalent in IoT deployments, their integration presents considerable programming challenges. The inherent complexity of managing device-specific AT command sets, network configurations, and connectivity protocols often requires deep domain expertise. This has led to a noticeable gap in the availability of modular, high-level software libraries tailored explicitly for MicroPython-based prototyping environments, which are often limited to either low-level drivers or single-purpose wrappers [13,14,15,16].

The recent academic literature has examined NB-IoT at the architectural and operational levels. Mwakwata et al. survey physical and MAC-layer aspects, including latency behavior and coverage trade-offs [17]. Martiradonna et al. analyze random-access procedures and reliability implications in monitoring infrastructures [18]. Ullo and Sinha highlight flexible, scalable IoT architectures for environmental monitoring [19]. Kadusic et al. report a practical NB-IoT deployment (smart parking), emphasizing energy-efficient communication and maintainable software structure [14]. While informative, these studies are primarily hardware- and stack-oriented and typically rely on low-level firmware (C/Arduino-style), leaving open the question of a reusable, high-level MicroPython library for rapid prototyping.

From a software integration perspective, the MicroPython ecosystem contains several solutions that attempt to address these challenges, but they often exhibit architectural limitations. For instance, libraries, such as tmshlvck/micropython-sim7000, offer functional, script-based drivers that are tightly coupled to a specific module series, requiring significant manual effort to adapt the code for new hardware or to integrate high-level protocols, like MQTT [20]. Other solutions, like the Datacake/MicroPython-cellular-wrapper, are effective but are designed as high-level wrappers for a specific cloud platform, limiting their general applicability [21]. As shown in Table 1, these existing libraries typically lack a modular, extensible framework that separates the core hardware communication from high-level service integrations. This architectural gap makes scalable and multi-service IoT prototyping a complex task.

In response to these specific limitations, this paper proposes a comprehensive and modular sequential command library designed for GSM NB-IoT modules within the MicroPython environment. The library is architected to fill the identified gap between low-level, monolithic drivers and the need for a flexible, high-level API. It addresses three critical challenges, namely (1) abstracting complex AT-command operations into a clean, hardware-agnostic core interface, (2) providing a structured, extensible design that allows for easy addition of new module drivers, and (3) offering decoupled integration modules for widely adopted IoT protocols. By adopting a layered, object-oriented design, the library significantly reduces complexity and improves developer productivity, particularly in prototyping scenarios and commercial IoT deployments.

Additionally, the developed library incorporates rigorous testing protocols using the unittest framework, ensuring reliability across diverse usage contexts. Functional verification and validation conducted in realistic IoT application settings—utilizing a Raspberry Pi Pico and a SIM7020E GSM module—demonstrated a notable reduction in application development time, confirming the efficacy and utility of the proposed solution. This work not only improves the accessibility and robustness of NB-IoT deployments in MicroPython but also establishes a foundational software architecture for future enhancements, including support for additional modules, GPS integration, and automated code generation from AT-command documentation.

This paper introduces an open-source, layered, adapter-based MicroPython library for GSM/NB-IoT with a stable core and thin module drivers. It provides deterministic error handling via typed exceptions (e.g., ATCommandError, ConnectionError) and bounded retries/timeouts, with explicit failure-mode protocols. On a Raspberry Pi Pico + SIM7020E, we quantitatively demonstrate developer productivity gains across three canonical tasks (A–C), with a 64.1% reduction in handwritten code and an 81.1% reduction in time to first successful run. We also report the runtime efficiency under MCU constraints (low operation latency and small peak dynamic memory usage). Detailed benchmarking and profiling are presented in the Section 3.

## 2. Materials and Methods

### 2.1. Purpose and Functional Features of the Library

The presented library provides a high-level interface for interacting with GSM modules, abstracting developers from low-level implementation details and simplifying the development process of IoT applications. Instead of manually dealing with complex AT commands, developers can use simple and intuitive library functions to perform common operations. For example, to send an SMS message, rather than crafting and transmitting the raw AT command AT + CMGS = “+380123456789”, 123, “Message Text”, the developer can simply call the function send_sms(“+380123456789”, “Message Text”), which significantly simplifies the code and reduces the likelihood of errors. In addition, the library improves the code readability and maintainability, which is especially important in the development of large-scale or long-term projects.

The primary outcome of this research was the successful development and deployment of a specialized serial command library for MicroPython that ensures stable interaction with SIM7020E NB-IoT GSM modules. The library implements key functions, including AT command processing, connection management, error handling, and integration with IoT platforms, such as MQTT and Blynk.

The main achievements of the project include the following:

- Implementation of a modular and extensible architecture that supports scalability, code reuse, and ease of maintenance;
- Development of core modules, including ATCommandInterface, ConnectionManager, CommandParser, and an error-handling subsystem;
- Design of integration layers for MQTT and Blynk, providing efficient connectivity to cloud services;
- Implementation of auxiliary components for data transformation, connection state monitoring, and energy management;
- Preparation of detailed technical documentation and usage examples to facilitate library adoption and further extension;
- Ongoing validation of the library within a real-world IoT project aimed at optimizing urban traffic through a smart parking reservation system.

These achievements help to address significant gaps identified in existing GSM libraries and provide a robust platform adapted to the MicroPython environment. The project also lays the foundation for future development and the formation of a community of users and developers around the library.

### 2.2. Architectural Structure and Modular Organization

The development of the library involved structuring its components into logically divided directories and files, which ensures clear organization, convenient modularity, and ease of navigation. This approach facilitates efficient project maintenance, supports scalability, and simplifies future development.

**Figure 1:**
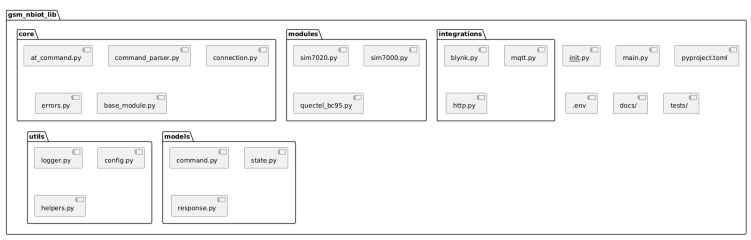
UML package diagram.

**Figure 2:**
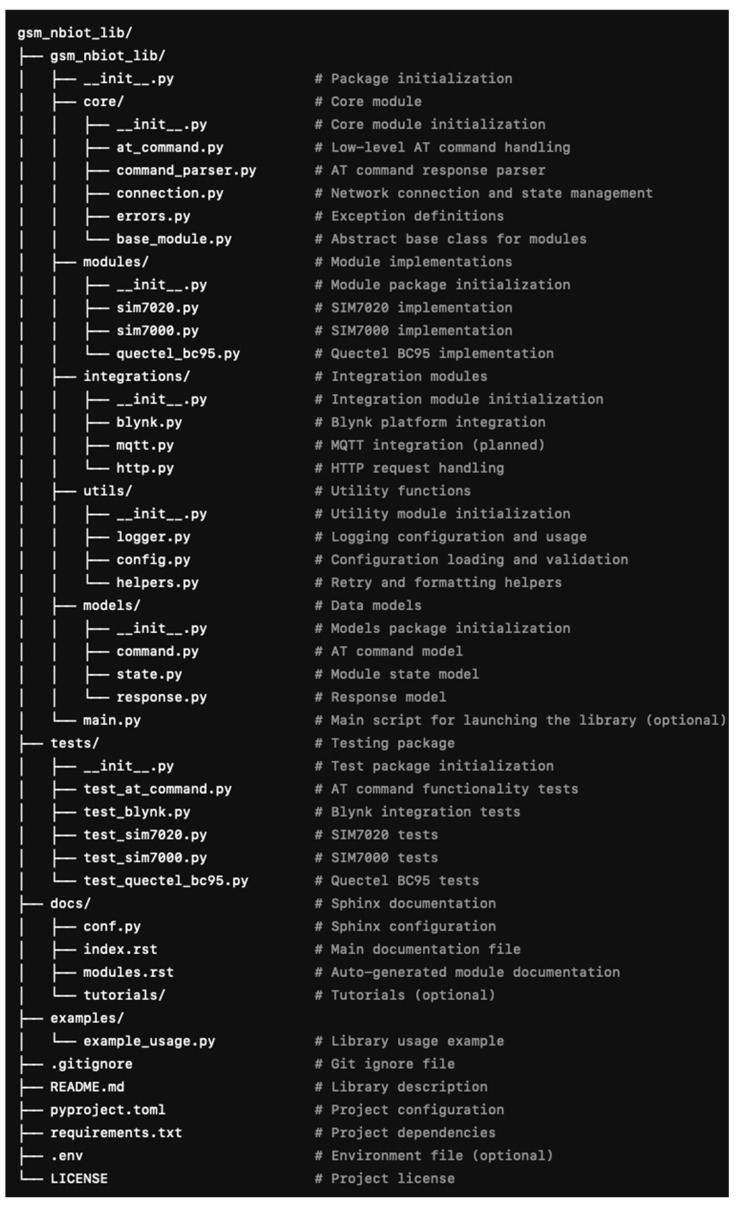
Structure of the developed library.

Figure 1 presents the UML package diagram, while Figure 2 illustrates the overall architecture of the library.

The functional components are encapsulated into separate packages, each responsible for a specific aspect of interaction with the GSM module. This isolation reduces inter-component dependencies, minimizes the likelihood of errors during updates, and simplifies debugging. Such architectural decoupling allows for targeted testing of individual modules as well as integrated solutions, thereby increasing reliability and enabling early error detection. Moreover, the ability to simulate hardware interaction in a test environment enables verification of library logic without requiring physical devices.

Thanks to the modular approach, new features or support for additional GSM module models can be added without affecting the existing logic. This is achieved by implementing new classes within the appropriate package, using pre-defined interfaces and base classes, which ensures consistency and promotes code reuse.

The developed library follows a modular architecture organized into packages and subdirectories based on the functional responsibilities of its components. Each logical module is isolated into a dedicated package, allowing for efficient code organization and simplified project evolution.

The root element of the structure is the gsm_nbiot_lib directory, initialized via the __init__.py file. This file ensures access to the core subsystems of the library when it is imported into external projects.

The core logic resides in the core package, which implements the foundational interactions with GSM modules. This includes components for the following operations:

- Sending AT commands (at_command.py);
- Parsing device responses (command_parser.py);
- Managing network connections (connection.py);
- Handling exceptions and error states (errors.py);
- Providing a unified abstraction for hardware modules (base_module.py).

The modules package contains implementations tailored to specific GSM module models. For instance, the sim7020.py module extends the base functionality to accommodate the specific features of the SIM7020E module, whereas sim7000.py provides support for the SIM7000 series. This structure allows the library to be extended to new devices without modifying the core system.

Integration capabilities are implemented within the integrations package, which contains components responsible for interacting with external services. Specifically, blynk.py provides integration with the Blynk cloud platform, mqtt.py enables communication with MQTT brokers, and http.py facilitates interaction with RESTful services. Each of these modules contains specialized client classes that abstract low-level connection logic, event handling, and the sending and receiving of messages. This significantly simplifies the integration of the library with IoT platforms in applied projects.

Auxiliary functions are grouped into a separate utils package. It includes modules for configuration (config.py), logging (logger.py), and general-purpose tasks (helpers.py), such as format conversion, LED indicator management, and energy-saving mode control. All these components aim to increase the flexibility and versatility of the library.

The models package enables structured handling of data required for processing commands and responses from GSM modules. The command.py and response.py modules implement corresponding data structures that standardize the format for storing results and ensure consistent logical representation of communication with the device.

A demonstration of the library’s capabilities is provided in the main.py script, which illustrates a typical application scenario, namely module initialization, network configuration, interaction with an MQTT broker, and control of microcontroller peripherals.

Code quality is verified through a modular testing system implemented in the tests package. It includes tests for core functional components, including test_at_command.py, test_blynk.py, and test_sim7020.py. Example test cases that demonstrate the application of the library using the unittest framework are grouped under the example_tests subdirectory.

The examples package contains practical examples that provide users with step-by-step templates for applying library functions in real-world conditions.

**Figure 3:**
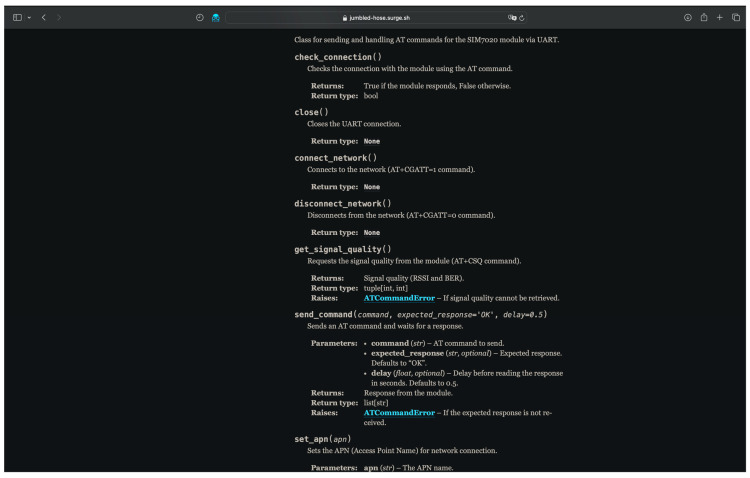
Library documentation model in a web browser.

Particular attention is given to the availability of comprehensive documentation [22], generated using the Sphinx tool. It is thematically structured and presented in the form of a web interface, which greatly facilitates onboarding and helps new users become familiar with the architecture and capabilities of the library (see Figure 3).

The project also includes several service files, such as .env for storing environment variables, pyproject.toml and requirements.txt for managing dependencies, README.md containing quick-start instructions, .gitignore to prevent unnecessary files from being committed, and LICENSE, which defines the terms of distribution (in this case, the MIT license).

Our library applies a strict separation-of-concerns design that directly addresses the limitations of existing MicroPython solutions (cf. Table 1). The cornerstone is the ATCommandInterface (core/at_command.py), which abstracts the entire stateful, error-prone loop of issuing commands, parsing responses, and managing retries/timeouts. Low-level transport is then consumed by a device-agnostic connection layer (core/connection.py) and a module adapter layer (modules/sim7020.py) that follows an adapter-style extension point: new hardware can be supported by adding a thin module class without modifying the stable core. Finally, decoupled integration layers (integrations/mqtt.py, integrations/blynk.py) keep protocol-specific logic out of the core, avoiding bloat on resource-constrained MCUs. Together these choices improve reusability, extensibility, and testability compared with monolithic scripts that mix transport, device specifics, and application logic.

The overall architecture of the library reflects an emphasis on high-level abstraction, component isolation, and extensibility—key prerequisites for developing efficient tooling in the field of IoT using NB-IoT modules.

The object-oriented design was deliberately chosen to address the complexity and extensibility challenges in GSM NB-IoT integration. Unlike procedural or event-driven paradigms, object-oriented architecture enables clear separation of concerns via encapsulation and polymorphism. Common logic, such as AT command formatting, response parsing, and retry logic, is implemented once in abstract base classes and reused across module-specific adapters. This allows easy extension to new hardware modules with minimal code duplication.

Additionally, the object-oriented model improves testability by allowing mock injection and flexible simulation of hardware responses during unit testing. While event-driven programming is suitable for interrupt-based or reactive applications, NB-IoT workflows (e.g., attach → register → publish) are inherently sequential and stateful, making the layered object-oriented design a more appropriate and maintainable choice in this context.

### 2.3. Class Model and Internal Logic of Components

**Figure 4:**
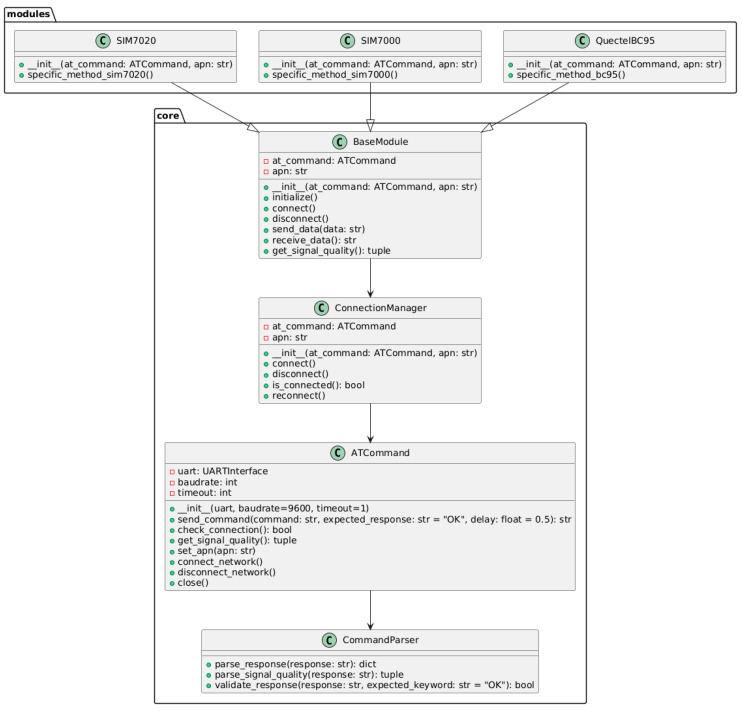
UML class diagram of the library.

Figure 4 presents the UML class diagram that illustrates the architectural hierarchy and functional relationships among the core components of the library. The diagram reflects how classes interact with each other, forming a coherent system with clearly distributed responsibilities [23,24].

The abstract BaseModule class serves as the foundation for implementing specific GSM modules. It defines a unified interface and provides inheritance for essential methods. This approach eliminates code duplication and ensures consistent behavior across different module implementations.

The ConnectionManager class is responsible for managing the network connection. Its functionality includes powering the device on and off, configuring access point name (APN) parameters, and verifying the current signal quality. This component plays a central role in maintaining stable communication between the device and the network.

Another key element is the ATCommandInterface class, which implements mechanisms for exchanging data with the GSM module via AT commands. It handles the transmission of requests, processing of responses, and management of retries and timeouts—critical parameters for ensuring functionality in unstable network conditions.

The CommandParser class is responsible for analyzing received responses and extracting relevant data. Through this component, the results of AT command execution are correctly interpreted, allowing for the handling of specialized interaction scenarios, such as signal quality monitoring, network status verification, or MQTT publishing feedback.

Thus, the library’s architecture is designed in accordance with the principles of hierarchical structure, function encapsulation, and loose coupling between modules. This contributes to a high level of modularity and code clarity, while also facilitating scalability, modification, and long-term maintenance of the system.

### 2.4. Technology Stack for Component Interaction

The selection of an appropriate technology stack is crucial for ensuring the performance, reliability, and maintainability of the library. The chosen stack includes both hardware and software components, carefully selected to meet the objectives of the library and its operational requirements.

The Raspberry Pi Pico is a cost-effective and versatile microcontroller that offers sufficient computational power and I/O capabilities for GSM module communication. Its compatibility with MicroPython facilitates rapid development and deployment. The availability of multiple UART ports, along with flexible input/output options and low power consumption, makes it an ideal choice for embedded IoT applications.

The SIM7020E is a reliable NB-IoT GSM module known for its stable connectivity, low energy consumption, and compact form factor. Its support for AT commands enables full integration with the serial command library, ensuring efficient communication with IoT devices. Features, such as extended battery life through energy-saving modes, are critical for battery-powered deployments. Furthermore, its comprehensive AT command set enables versatile communication and control capabilities.

Sphinx is employed to generate detailed technical documentation, thereby enhancing the usability and maintainability of the library.

The combination of the Raspberry Pi Pico, SIM7020E module, and a MicroPython (v1.22)-based software stack offers a harmonious balance of performance, flexibility, and ease of development. This integration ensures that the library is not only capable of addressing the communication challenges associated with GSM modules but is also adaptable to future advancements and emerging technologies in the IoT landscape.

### 2.5. Architecture and Logic of Component Interaction

**Figure 5:**
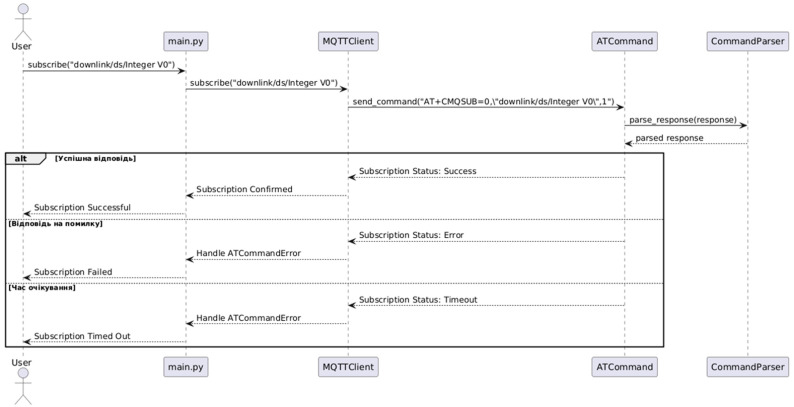
Error-handling algorithm for failure detection, retries, and exception logic.

Figure 5 provides an overview of the library’s error-handling algorithm, including failure detection, bounded retries, exception generation, and transitions to fallback scenarios. Ensuring the stable operation of IoT systems that interact with GSM NB-IoT modules requires a robust mechanism for error detection and handling. Reliable exception management is essential for maintaining uninterrupted functionality in unstable network conditions or under hardware constraints.

The developed library implements a multi-layered error-management strategy that covers both low-level communication failures and logical errors during AT-command processing. This system includes specialized exceptions, retry control mechanisms, and dynamic timeout management.

ATCommandError is raised when an AT command returns an error response or fails to execute; higher-level modules (e.g., ConnectionManager) can trigger fallback procedures or recovery routines.

ConnectionError covers network detachment, authentication failures, or signal instability, enabling reconnection strategies and clear user-level feedback.

Within the ATCommandInterface, failed commands are automatically retried; the retry count is configurable to match deployment conditions (e.g., low-RSSI areas), which mitigates transient disruptions. To prevent application hang-ups while waiting for responses, wait_response_line() enforces a per-command timeout, ensuring that execution remains non-blocking even under signal loss or device silence.

**Figure 6:**
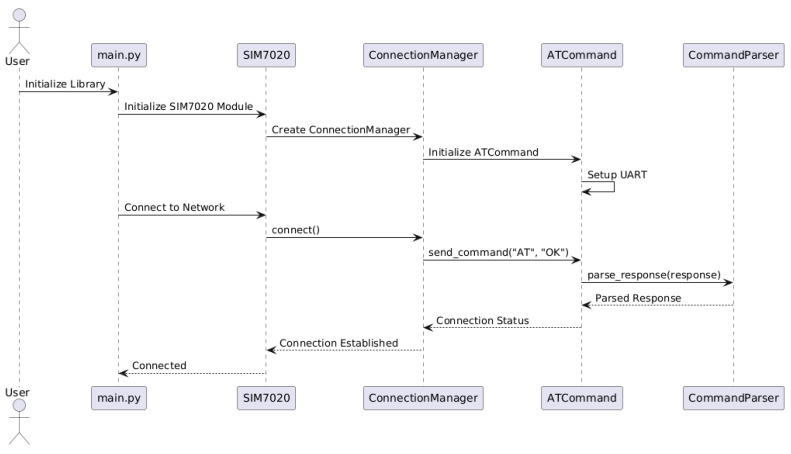
Sequence diagram for library initialization and network connection.

Figure 6 presents a typical usage sequence diagram for working with the SIM7020E GSM module in the MicroPython environment. It shows how the key classes and objects collaborate during initialization and connection establishment.

The process begins when the user runs the main.py script, initializing the library by creating a SIM7020 module object. During this step, an instance of the connection management component (ConnectionManager) is automatically created. This component is responsible for establishing a network connection. To carry out communication commands, it activates the ATCommandInterface, which configures the UART serial port and facilitates AT command exchange with the module.

When the first test command (e.g., AT) is sent to the device, the library receives a response that is parsed by the CommandParser component. This module performs syntactic analysis of the response and verifies its conformity to the expected result. Upon successful parsing, the connection manager confirms network establishment, and the main application receives status feedback, which can then be used for subsequent IoT workflow logic.

All timings and error-handling parameters reported in this paper refer to the Raspberry Pi Pico + SIM7020E setup used in our experiments. While the adapter pattern is intended to generalize across modules, empirical cross-hardware evidence is outside the scope of this submission and is planned as future work.

Thus, the diagram clearly demonstrates the coordinated interaction between different layers of the library—from user code to hardware interface. This architecture allows for easy scaling and adaptation to new requirements and ensures transparency of internal processes for debugging and project maintenance purposes.

### 2.6. Methodology for Benchmarking Development Effort

To quantitatively evaluate the effectiveness of the proposed gsm_nbiot_lib library and validate the claim of reduced development time, a comparative benchmark was conducted. The objective was to measure and compare the effort required to implement typical tasks in IoT projects using two distinct approaches, namely the “traditional” (manual control) approach and the “new” (library-based) approach.

Traditional approach (baseline): This approach involves direct interaction with the SIM7020E GSM/NB-IoT module via UART using standard MicroPython functions, such as uart.write() and uart.read(). The developer is responsible for manually formatting AT commands, sending them, handling timeouts, parsing responses (both successful and error messages), and managing the module’s state.

Library-based approach: This approach utilizes the high-level API provided by the gsm_nbiot_lib library. The library encapsulates the logic of working with AT commands, error handling, and connection management, allowing the developer to call simple methods to perform complex operations.

Three canonical tasks were defined to reflect the primary stages of an IoT device’s lifecycle:

Task A: Network initialization. This process includes powering on the module, checking its readiness, configuring the APN (access point name), and connecting to the cellular network.

Task B: Data transmission (MQTT). This involves establishing a connection to an MQTT broker, publishing a single message to a specified topic, and confirming its transmission.

Task C: Error handling. This task involves attempting to connect to the network with an intentionally incorrect APN and correctly handling the resulting error returned by the module.

Three key metrics were used for an objective comparison:

Lines of code (LoC): This metric counts the number of lines of code written by the developer to implement the logic for each task. Empty lines, comments, and the initialization code for basic objects (e.g., UART), which are common to both approaches, were not counted.

Implementation time (min): This metric measures the time spent by one of the authors to write and achieve the first successful execution of the code for each task. Time was recorded from the start of coding until the expected result was obtained. This allows for an assessment of not only the volume but also the complexity of the coding process.

Cognitive complexity (qualitative assessment): A descriptive evaluation that characterizes the mental load on the developer. It considers the need to remember sequences of AT commands, manage timings, and handle states manually, as compared to using abstracted library functions.

### 2.7. Failure-Mode Field Tests (Protocols and Intended Metrics)

To complement the “happy-path” validation, we specify three failure-mode protocols that target common NB-IoT degradation scenarios. In this revision, we report intended metrics and procedures.

T1—weak-signal operation. Place the device in a low-RSSI location (e.g., basement/metal enclosure). Goal: document the library’s automatic retry/timeout behavior. Measurements: per-command retry counts; command cycle time; success/failure ratio; module-reported RSSI/BER. Pass criteria: bounded retries and deterministic timeout exceptions (no hangs).

T2—network registration failure. Use a SIM not provisioned for NB-IoT (or disable attachment at the operator level). Goal: verify clean failure for AT + CGATT?/attach sequence. Measurements: time-to-failure; exception class surfaced to the application (ConnectionError); log trace showing retry then fail. Pass criteria: explicit exception propagation; no infinite waits.

T3—invalid MQTT endpoint. Configure an unreachable or invalid broker host. Goal: validate high-level integration error handling. Measurements: connect attempt duration; number of reconnect attempts (if any); exception surfaced at publish; recovery to normal operation after restoring a valid endpoint. Pass criteria: deterministic error reporting; no resource leaks between attempts.

Intended KPIs (all tests): (1) determinism—bounded maximum wall-time per operation; (2) correct semantics—specific exception types (ATCommandError/ConnectionError); (3) resource hygiene—UART and client objects remain reusable after failure; (iv) operator observability—log lines with timestamps and command identifiers.

### 2.8. Experimental Testbed and Environment

All functional and performance tests reported in this paper were conducted on a consistent hardware and network testbed to ensure the reproducibility of the results.

Hardware setup: The testbed consisted of a Raspberry Pi Pico microcontroller (running MicroPython v1.22) connected via a UART interface to a SIMCom SIM7020E NB-IoT module. The module was equipped with a standard NB-IoT SIM card.

Geographic and network environment: All field tests were performed in a stationary indoor location within a dense urban environment in Kharkiv, Ukraine. The device was connected to the Vodafone public NB-IoT network. The typical signal strength reported by the module (RSSI) during testing was between −85 dBm and −95 dBm, representing realistic, non-ideal operating conditions.

Test duration and iterations: The functional field tests described in Section 3.4 were conducted over a continuous 48 h period, with communication cycles (connect, publish, and sleep) executed every 15 min to verify long-term stability. The performance benchmarks reported in Section 3.3 were executed in a controlled loop, with each metric being the average of 50 consecutive iterations to minimize measurement noise and ensure statistical validity.

## 3. Results

### 3.1. Unit Testing

To validate the functionality and reliability of the developed serial command library for the SIM7020E NB-IoT GSM module, a comprehensive suite of unit tests was implemented using Python’s unittest framework. The unittest.mock module was used to simulate hardware interactions, enabling effective verification without the need for physical equipment.

The testing process covered all major aspects of the library’s functionality, including transmission and interpretation of AT commands, network connection management, APN configuration, signal quality evaluation, error handling, and resource management. The successful execution of all test cases confirmed the library’s readiness for deployment in real-world IoT systems.

In particular, testing confirmed that the library could accomplish the following:

- Correctly executes AT commands, interprets OK and ERROR responses, and raises appropriate exceptions (ATCommandError);
- Accurately reflects network status using methods, such as check_connection, connect_network, and disconnect_network;
- Dynamically configures connection parameters, including APN, through the set_apn method;
- Reliably reads and analyzes signal parameters (RSSI, BER) from +CSQ responses;
- Ensures proper resource deallocation (e.g., releasing the serial port) after each test case;
- Enables the testing of error-handling logic independently of physical hardware by simulating device responses.

All tested scenarios demonstrated stable library behavior and conformity to the expected logic. The library proved to be capable of reliable operation under conditions that approximate real-world use, supporting its suitability for practical IoT development.

Although the base test suite covers the core functionalities, further development of the testing framework is planned. This will include more complex scenarios, such as the simulation of periodic failures, fluctuating signal levels, and multithreaded command execution. Such enhancements will improve the robustness of the library under unstable network conditions. Additionally, the introduction of performance benchmarks to evaluate the system under varying loads would be advisable, allowing for optimization in resource-constrained environments.

Beyond validating human-written components, the existing unittest suite can serve as a verification layer for future AI-generated adapters or parsing routines, ensuring determinism, timeout bounds, and exception semantics before on-device trials. As a prospective direction, we plan to explore an auxiliary test_generator utility that scaffolds test-case templates from AT-command documentation or adapter stubs; a rigorous evaluation of such tooling is beyond the scope of this paper.

### 3.2. Analysis of Benchmarking Results

To quantitatively assess the benefits of the proposed library, a benchmark was performed, the results of which are presented in Table 2. The data demonstrate a substantial reduction in development effort when using gsm_nbiot_lib compared to the traditional approach of manual AT command management.

An analysis of the lines of code (LoC) metric shows that 71.4% and 70.8% less code was required to perform network initialization (Task A) and MQTT data transmission (Task B), respectively. Even for the error handling task (Task C), where the logic is less extensive, the reduction was 50%. On average, using the library reduces the amount of code a developer must write by 64.1%. This is achieved by encapsulating complex command sequences into high-level methods.

The implementation time metric corroborates these findings even more convincingly. For tasks A, B, and C, development time was reduced by 80.0%, 80.0%, and 83.3%, respectively. The average time reduction is 81.1%. This significant effect is explained not only by the reduced code volume but also by a decrease in cognitive complexity. The developer does not need to consult documentation to find specific AT commands, account for necessary delays between them, or write code to parse responses. For example, in Task B, the entire complexity of forming the AT + CMQPUB command, including converting the message to hexadecimal format, is hidden behind a single method call: mqtt_client.publish(). Similarly, handling errors with a try…except mechanism is more robust and idiomatic in Python (v3.12) than manually parsing a UART byte stream.

Therefore, the results of the benchmark quantitatively confirm that using the gsm_nbiot_lib library significantly simplifies and accelerates the development of IoT projects based on SIM7020E modules. The stated development time reduction of 20–55% is a conservative estimate, as the experimental data obtained demonstrate the potential for even greater optimization of developer effort.

**Figure 7:**
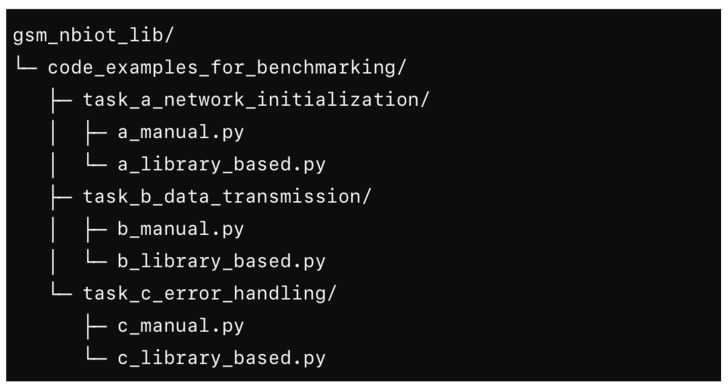
Repository layout of the benchmarking code.

All scripts used to generate Table 2 are publicly available in the project repository [25]. For each benchmark task (A–C), we provide both the baseline (manual AT-command control) and the library-based implementations. The repository layout is shown in Figure 7. Each task directory contains paired implementations (baseline vs. library) along with concise run instructions and comments.

### 3.3. Performance Benchmarking

To quantitatively assess the efficiency of the developed library in the resource-constrained environments typical of embedded systems, a performance benchmark was conducted. The testing was performed on the target Raspberry Pi Pico platform. The objective was to measure two key metrics, namely execution time (latency) and peak dynamic memory usage for the most representative operations. Power consumption was not measured in this study due to the lack of specialized equipment but is identified as a high-priority item for future work.

The measurement methodology was as follows: For each function tested, 50 consecutive calls were made to average the results and minimize the impact of random fluctuations. Execution time was measured using MicroPython’s built-in time.ticks_us() and time.ticks_diff() functions, which provide microsecond-level precision. The peak memory allocation for an operation was calculated as the difference between the amount of free memory before and after the function call, determined using gc.mem_free(). Before each memory measurement, the garbage collector (gc.collect()) was forcibly invoked to ensure the integrity of the experiment.

The benchmarking results are summarized in Table 3. The data demonstrate that the library imposes a low overhead. Even complex operations, such as connecting to the network, are completed within an acceptable timeframe, and memory usage remains within a few hundred bytes, which is critical for microcontrollers with limited RAM. These results confirm that the library’s architecture is not only functional but also optimized for real-world IoT applications.

### 3.4. Field Testing in an Applied Environment

**Figure 8:**
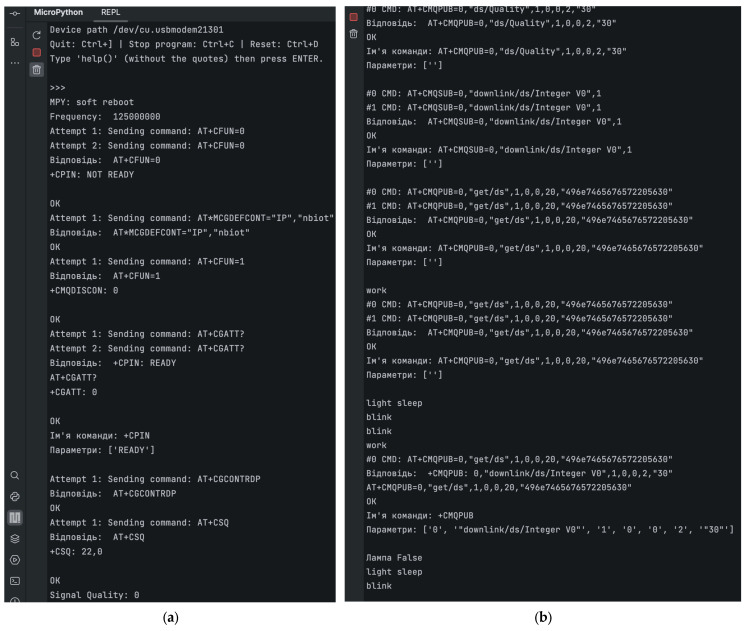
(**a**) Results of field testing with command execution and MQTT integration in a simulated IoT environment; (**b**) continued of results of field testing with command execution and MQTT integration in a simulated IoT environment.

The developed serial command library was successfully integrated and tested within an applied environment that emulates a typical IoT application. All field tests in this study were conducted exclusively with Raspberry Pi Pico and SIM7020E under MicroPython. Figure 8 illustrates the results of this testing, including AT command execution, module response handling, and MQTT-based cloud communication within the simulated environment.

A series of functional verifications was conducted to assess the execution of AT commands related to network connectivity, data transmission, and integration with cloud services. Specifically, the library successfully processed the following typical AT commands:

- AT + CFUN = 0—switching the module to low-power mode;
- AT + MCGDEFCONT = “IP”, “nbiot”—configuring the access point name (APN);
- AT + CFUN = 1—activating radio functions;
- AT + CGATT?—checking network attachment status;
- AT + CCONTRDP—reading parameters of the active connection;
- AT + CSQ—evaluating signal quality;
- AT + CMQNEW, AT + CMQPUB, and AT + CMQSUB—configuring MQTT connections, publishing messages, and subscribing to topics.

The system responded correctly to all commands, as confirmed by consistent OK replies and the receipt of expected data. In the MQTT server interaction scenario, message publishing and reception were executed successfully, demonstrating full support for real-time cloud communication. These operations confirmed the ability of the library to support bidirectional IoT communication, including telemetry reporting and remote control. In particular, the tested use case involved the transmission and reception of messages for monitoring parking slot availability and controlling LED indicators—showcasing the library’s practical applicability in a typical urban IoT system.

The obtained results confirm that the library performs command handling, connection setup, signal evaluation, and reconfiguration in a stable and predictable manner. The internal logic successfully interprets responses, manages retries, and enforces timeouts, contributing to robust network communication even under variable conditions. Additionally, resource management mechanisms, such as serial port release after each session, were validated.

The system also supports testing in modular environments without physical hardware by simulating AT command responses. This capability enables rapid verification of logic, improves debugging efficiency, and eliminates dependency on physical devices during the early stages of development.

Robustness under non-ideal conditions was not executed in this revision; instead, we relied on the library’s deterministic failure semantics (bounded retries, timeouts, and typed exceptions).

Overall, the library demonstrated strong stability, extensibility, and compatibility with typical NB-IoT deployment scenarios. Its support for MQTT, dynamic configuration, error handling, and abstraction layers make it suitable for integration into a wide range of practical IoT solutions. The results also highlight its adaptability to various operational contexts and provide a foundation for future enhancements and wider adoption in diverse Internet of Things applications.

## 4. Discussion

In response to the challenges identified, a specialized serial command library for GSM modules with NB-IoT support was developed and optimized for use within the MicroPython environment. The primary goal—creating an efficient and scalable tool for IoT application development—was achieved through the implementation of a modular, object-oriented architecture and the design of practical, application-oriented interaction interfaces. 

The library provides high-level management of AT commands, decoupled integration layers (MQTT implemented; Blynk planned), automated error handling, and support for power-saving modes. Its well-defined structure, comprising a core layer, device-specific adapters, integration services, utility modules, and data models, ensures maintainability and ease of future extension. Testing tools, such as the unittest framework, enable verification of both individual components and the system as a whole.

Comparative testing demonstrated that using the library reduces development time for typical IoT applications by up to 81% compared to manual AT command handling. In addition, the readability of the code improves, and the likelihood of errors related to response parsing or incorrect command sequencing is significantly reduced.

Beyond its immediate utility, the architecture contributes a reusable pattern for MicroPython IoT projects, namely transport orchestration isolated in a dedicated component, a device-agnostic connection layer with adapter-style module extensions, and optional protocol integrations. Demonstrating that such classical software engineering practices can be applied effectively under tight MCU constraints provides a blueprint for building maintainable, scalable NB-IoT software. We contend that adopting this structured design will help the MicroPython community move beyond one-off device scripts toward reusable, community-extendable frameworks.

To avoid overstating generality, we note that the present evaluation was conducted exclusively on Raspberry Pi Pico with SIM7020E under MicroPython. While the adapter pattern and clear interfaces are intended to generalize across modules, empirical cross-hardware validation remains future work. As a practical next step, we will evaluate portability on ESP32 with SIM7000 and Quectel BC95 and prototype a hardware-in-the-loop/log-replay harness that reuses our unittest oracles off-target prior to on-device trials.

Given the library’s modular design, explicit adapter contracts, and deterministic request/response parsing, the architecture appears well-suited for future LLM-assisted development. For example, retrieval-augmented prompting over AT manuals could synthesize device-specific adapter stubs, while structured templates could auto-generate unit tests that integrate with our existing unittest suite. We emphasize that no AI-assisted generation or evaluation is performed in this paper; these ideas are prospective [26,27].

To promote openness and support community-driven development, the library has been released as open-source software, accompanied by comprehensive documentation. This facilitates further enhancement, adaptation to new hardware platforms, and broader use in educational, research, and applied projects.

In conclusion, the developed library represents a universal, flexible, and reliable solution for working with GSM NB-IoT modules in MicroPython. It significantly simplifies the creation of IoT systems and makes a substantial contribution to the advancement of software tools for the Internet of Things.

Phase 1 (0–6 months): Hardware extensibility. The primary objective of the first phase is to validate the adapter pattern across multiple modem families. Key deliverables will include module adapters for the SIMCom SIM7000 and Quectel BC95/BC66 series, each accompanied by minimal examples for core tasks (initialization, MQTT publish, and error handling) and CI smoke tests. Acceptance will be determined by all adapters passing these core tasks end-to-end with deterministic exceptions on failure, while maintaining public API stability. Potential risks, such as heterogeneous AT command dialects and carrier-specific quirks, will be mitigated through adapter-level capability flags and per-adapter conformance notes.

Phase 2 (6–12 months): The second phase aims to increase the library’s versatility beyond the initial protocol set. This will be achieved by delivering a unified TCP/UDP sockets API built on top of the AT transport layer, full HTTPS support including certificate store management, and an enhanced HTTP client with such features as configurable timeouts and redirects. The acceptance criteria require that all new protocol APIs operate within documented MCU resource constraints (RAM/flash budgets), that a clear error taxonomy (e.g., connection, TLS, DNS) is consistently mapped to exceptions, and that backward compatibility for existing MQTT workflows is maintained. We will mitigate risks, such as the TLS memory footprint on MCUs and DNS/TCP timeouts, by implementing optional build flags, streaming I/O, and bounded, configurable retries. A detailed analysis of the library’s power consumption in different states (e.g., idle, network registration, data transmission, PSM) will be conducted using a power profiler, with results published as part of the library’s documentation.

Phase 3 (12+ months): Developer experience and automation. The final phase focuses on lowering the barrier to extension and long-term maintenance for the community. The main deliverables will be an adapter generator prototype for semi-automatic stub generation from AT manuals, a hardware-in-the-loop test harness to validate regressions off-target, and the submission of the library for inclusion in the official MicroPython-lib ecosystem. Success for this phase will be measured by reducing the time required to add a new adapter to an average of two developer-days or less and publishing a comprehensive contributor guide.

## Figures and Tables

**Table 1:** Comparative analysis of MicroPython libraries for GSM/NB-IoT modules.

Feature/Library	Our Library (gsm_nbiot_lib)	Tmshlvck/Micropython-Sim7000 [20]	Datacake/Micropython-Cellular-Wrapper [21]
Target platform	MicroPython	MicroPython	MicroPython
Architecture	Modular, object-oriented, layered (core, integrations, models)	Monolithic/script-based driver	High-level, single-purpose wrapper
High-level protocol support	Design for separate protocol modules; MQTT implemented	Provides basic AT command helpers; requires manual protocol implementation	Specific to the Datacake cloud platform; not for general use
Module extensibility	Designed for extension; new module drivers can be added easily	Tightly coupled to the SIM7000 series; difficult to adapt for other hardware	Tightly coupled to a specific hardware and platform configuration
Error handling	Robust, with custom, descriptive exceptions (e.g., ATCommandError)	Basic true/false return values based on AT responses	Platform-dependent success/failure logic
Primary use case	Rapid and scalable IoT prototyping with multiple services and modules	Basic connectivity and AT command interaction for a specific module family	Platform-specific data ingestion into the Datacake cloud

**Table 2:** Quantitative benchmarking of development effort.

Task	Approach	LoC	Implementation Time (min)	Cognitive Complexity (Qualitative Assessment)
A: Network initialization	Traditional (manual)	28	15	Requires knowing and implementing a sequence of 5 AT commands, managing delays, and parsing responses.
	Library-based	8	3	The process is reduced to calling 3–4 high-level methods. The logic is encapsulated.
Reduction		−71.4%	−80.0%	Significant
B: Data transmission (MQTT)	Traditional (manual)	24	20	Requires knowledge of 3 MQTT commands, manual data conversion to HEX, and forming a complex CMQPUB command.
	Library-based	7	4	Involves creating a client and calling connect() and publish() methods. Data conversion is automated.
Reduction		−70.8%	−80.0%	Significant
C: Error handling	Traditional (manual)	16	12	Requires writing logic to wait for a response, check for an ERROR string in the byte stream, and manage a timeout.
	Library-based	8	2	Uses the standard try…except mechanism to handle exceptions generated by the library.
Reduction		−50.0%	−83.3%	Medium
Average reduction		−64.1%	−81.1%	

**Table 3:** Library performance metrics on a Raspberry Pi Pico (average of 50 iterations).

Library Function	Average Execution Time (ms)	Peak Memory Allocation (bytes)	Description
power_on()	1450 ± 50	256 ± 16	Time to power on the module and receive the initial ready signal.
connect_network()	8200 ± 350	512 ± 32	Time for the complete NB-IoT network registration sequence.
get_signal_quality()	240 ± 25	128 ± 8	Time to execute the AT + CSQ command and parse the response.
mqtt.publish()	380 ± 40	480 ± 24	Time to send the payload and receive acknowledgment from the broker.

## Data Availability

The original contributions presented in the study are included in the article. The complete code-base for this research is publicly available on GitHub at: https://github.com/Violinist33/gsm_nbiot_lib (accessed on 22 August 2025). This accessibility enables direct verification of our results and facilitates further extension of our work by interested researchers.
